# Experimental Analysis and Parametric Optimization on Compressive Properties of Diamond-Reinforced Porous Al Composites

**DOI:** 10.3390/ma16010091

**Published:** 2022-12-22

**Authors:** Bisma Parveez, Nur Ayuni Jamal, Abdul Aabid, Muneer Baig, Farazila Yusof

**Affiliations:** 1 Department of Manufacturing and Materials Engineering, Kulliyyah of Engineering, International Islamic University Malaysia, Kuala Lumpur 53100, Malaysia; 2 Department of Engineering Management, College of Engineering, Prince Sultan University, P.O. Box 66833, Riyadh 11586, Saudi Arabia; 3 Department of Mechanical Engineering, Faculty of Engineering, Centre of Advanced Manufacturing & Material Processing (AMMP Centre), Universiti Malaya, Kuala Lumpur 50603, Malaysia

**Keywords:** porous aluminum composite, porosity, relative density, plateau stress, energy absorption capacity, Taguchi L_9_ orthogonal array

## Abstract

The present study aims to optimize the compressive properties of porous aluminum composites fabricated using the powder metallurgy (PM) space holder technique. These properties were optimized by taking into consideration different processing factors such as sintering temperature, compaction pressure, and sintering time. The experimental design was formulated using L_9_ orthogonal array by employing these three parameters at three levels. The density, porosity, plateau stress, and energy absorption capacity were determined and analyzed. The impact of individual input parameters was evaluated using the Taguchi-based S/N ratio and analysis of variance (ANOVA). The main effect plots outlined the optimum parameter levels to achieve maximum values for compressive properties (plateau stress and energy absorption capacity). The results revealed that the sintering temperature and time significantly impact compressive properties. The ANOVA analysis exhibited similar results, with maximum contribution from sintering temperature. Further response optimization of compressive properties concluded that the maximum values could be achieved at optimum parameters, i.e., a sintering temperature of 590 °C, compaction pressure of 350 MPa, and sintering time of 90 min. Further, confirmation tests on the optimized parameters revealed improved results and some minor errors and deviations indicating that the selected parameters are vital for controlling the compressive properties of the aluminum composites.

## 1. Introduction

Porous composites are low density materials with unique properties, suitable for lightweight structures, thermal management, and energy absorption applications. Due to the broader accessibility of practical technologies and a better understanding of their physiological, chemical, and mechanical characteristics, these materials have gained increasing attention in recent decades [1]. Conceptually, cell connectivity categorizes cellular metals as closed or open-celled. Many metal composites, such as derivatives of aluminum (Al) [2,3,4], magnesium (Mg) [5,6], and titanium (Ti) [7,8,9], have been foamed or formed into cellular structures. Because of their ease of fabrication, Al composites are the most popular metal composites used for developing metal foams [10], whereas tin (Sn) and Mg are used as additives to aid the Al matrix in liquid phase sintering [11]. Furthermore, no intermetallic phases are formed between Al and Sn as they are mutually insoluble in the solid phase. However, the immiscibility of both metal liquids leads to an ideal liquid-phase sintering system [12]. Additionally, when Mg powder is added to the mixture, the wetting angle decreased, and the liquid flows into micropores by capillary action. The liquid fills the pores during the liquid-phase sintering, resulting in a high-density foam framework [12,13]. Additionally, boron has been used in stainless steel to improve its mechanical properties and microstructure. It is another active sintering additive used in metal composites. At temperatures higher than the eutectic transformation temperature, the eutectic reaction occurs between metals and boron, resulting in liquid phase formation, which helps to sinter the composites [14]. When dispersed in the stainless-steel matrix, boron can form metal-boride complexes and separate at grain boundaries [15]. Previous studies have reported the addition of silicon carbide (SiC) [16,17], alumina (Al_2_O_3_) [18,19], and carbon nanotube (CNT) [20,21,22] to porous Al composites. The SiC particle-reinforced Al composite foams exhibited higher compressive stress but are more brittle than pure Al foams [23]. In addition, in the Al/SiO_2_ composite foam fabricated by Salehi et al. [24], there was an increase in the plateau stress and energy absorption with the addition of SiO_2_ up to a certain limit, beyond which it decreased. A powder metallurgy-based porous Al composite reinforced with SiC was found to exhibit brittle compression behavior [25]. Daoud et al. [26] developed an Al_2_O_3_ reinforced A359 alloy matrix porous composites resulting into a roughly equiaxed polyhedral cell structured microstructure. These alumina particles were distributed uniformly, leading to increase in plateau stress, yield stress, plastic stress, young’s modulus, and energy absorption with an increase in alumina content. The B_4_C-reinforced Al-foams were developed via powder metallurgy-space holder technique; the yield strength and energy absorption improved on addition of B_4_C [4]. In the past few years, high-specific strength and lightweight metal matrix composites reinforced with CNTs have become the research focus [27]. The challenges in preparing high-quality composites include their clustering tendency, poor wettability, and large discrepancy in density and size between CNTs and metal matrix composites [28]. Several techniques, such as high-energy ball milling [29,30], pretreatment of CNTs [31], and CNTs coating, have been used to improve the distribution of CNTs and their interfacial bonding with metal matrix composites. Cu-coated carbon-fiber-reinforced porous Al composites were developed showing good wettability and uniform distribution of coated CNT particles, and as a result exhibited improved collapse stress. The Ni-coated carbon-fiber-reinforced porous Al composites fabricated using the powder metallurgy technique revealed an enhanced wettability at the interfaces and effective distribution of the coated CNTs in the Al matrix [32].

The porosities in porous Al composites have been achieved by researchers using blowing agents such as calcium carbonates [33,34] and titanium hydride (TiH_2_) [35], or space holders such as sodium chloride (NaCl) [2,36] or carbamide [20,37]. The space holders have proven to be more efficient in tailoring the shape and size of porosities, as pores usually replicate the shape and size of space holders in these porous composites. However, any reaction between the residue of space-holding particles and the metal matrix may impair the mechanical properties of the resulting composite [5]. Thus, an alternative space holder material with the most negligible affinity with metals, such as poly (methyl methacrylate) (PMMA), has been explored [38]. The closed-cell porous Al composites with varying porosities and densities were produced using different amounts of PMMA particles, as a result the porosities were efficiently regulated [39]. Additionally, the Mg-based porous composites were developed using spherical PMMA particles as space holders. The results revealed the formation of spherical pores that replicate the shape and size of PMMA particles, and thus have better control of porosity [40].

The fabrication of these porous Al composites using space holders has been carried out efficiently using a powder metallurgy technique (PM). It is one of the most diversified and constantly evolving metalworking methodologies for fabricating various shapes and sizes. The main benefit of PM involves producing parts with high dimensional accuracy and high productivity by involving limited use of materials and energy, as a result there occurs minimal waste of raw materials [41]. The key processes include mixing, compaction, and sintering. Sintering temperatures are important in determining the mechanical properties of porous composites as they determine the bonding between metal matrix particles and reinforcements [42]. Despite research on porous Al composites, the study on optimizing composite properties based on parameters in the powder metallurgy process is still limited [43,44]. According to the literature, treated carbonaceous reinforcements can be used as a potential reinforcement material to strengthen Al foams. Notably, the use of diamond in strengthening porous Al composites has not been reported yet, mainly due to its poor interfacial bonding with the metallic matrix.

In this study, the powder metallurgy process with varying parameters was employed for fabricating porous Al composites using nine sets of parameters acquired from the L_9_ orthogonal array. These composites were reinforced with Ti-coated diamond particles and PMMA particles were used as space holders. The morphology, density, porosity, and compressive properties of porous Al composites were investigated. Finally, using statistical analysis, the results were predicted and compared with experimental results to find the deviations, followed by finding the optimum processing parameters and confirming the compressive property improvement at these optimized parameters.

## 2. Methodology

### 2.1. Experimental Procedure

#### 2.1.1. Preparation of Porous Al Composites

Al, Mg, Sn, B, and Copper (Cu) powder acquired from Nova Scientific Resources Sdn Bhd with particle size and purity as mentioned in Table 1 were used in this study. Mg, Sn, Cu, and B powders were mixed with Al powder to promote the formation of a liquid phase during the sintering process [10,11]. The powder was used to produce the main framework of the porous composite.

**Figure 1:**
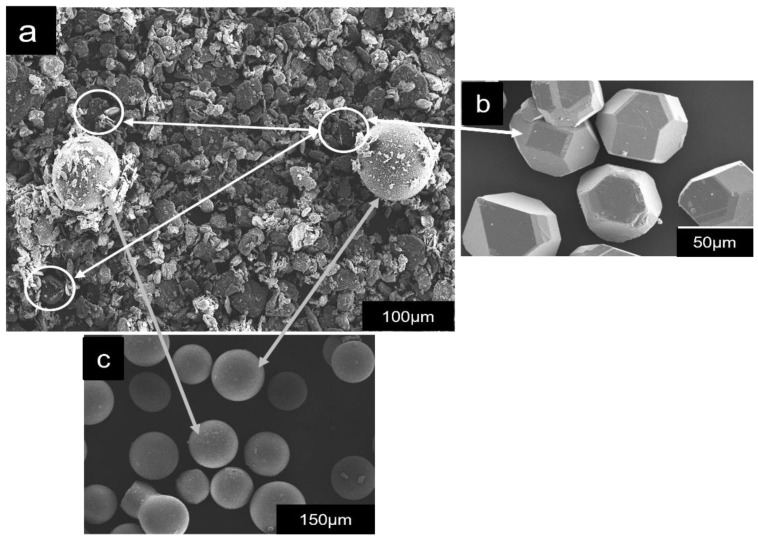
SEM micrography of (**a**) composite powder mix, (**b**) Ti-coated diamond particles, and (**c**) PMMA particles.

Titanium (Ti)-coated diamond was used as reinforcement material, and spherical PMMA particles were selected as space holders with an average particle size of 45 and 150 µm, respectively. The PMMA had a density of 1.33 g/cm^3^ and thermal decomposition temperature of 360–400 °C. The powders were mixed in three steps, as demonstrated in Table 2, resulting in uniformity in the mixture as shown in Figure 1a.

Firstly, metallic powders were mixed for 12 h at 300 rpm in a horizontal ball mill to prepare matrix alloy mix with the ball-to-powder ratio of 10:1. Then, Ti-coated diamond particles were mixed with the metallic mix using an oscillating mixer for 2 h at 800 rpm. The resultant powder was finally mixed with PMMA particles for 2 h using an oscillating mixer at 800 rpm to get a uniform distribution of diamond particles and PMMA particles in the metallic mix as evident from Figure 1. Before mixing, PMMA particles were mixed with CLE-safe oil as a binder to create a sticky surface for metallic mix adhesion as shown in Figure 1a. This step also prevented segregation during processing and promoted the uniform distribution of PMMA in the porous Al composites.

**Figure 2:**
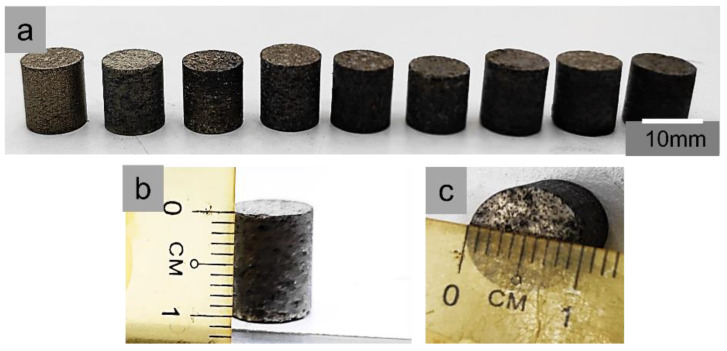
Sintered samples of, (**a**) 10 mm × 10 mm (1 cm × 1 cm) size, (**b**) 1 cm height, (**c**) 1 cm diameter.

After the mixing process, compaction of the final powder mixture was carried out at three different compaction pressures, viz., 350, 380, and 400 MPa using a hydraulic compaction machine. Mixed powders were compressed in the die with a diameter of 10 mm to obtain samples with a size of approximately 10 mm × 10 mm, as shown in Figure 2. Sintering was carried out on the compacted specimen to impart strength and integrity. Samples were sintered at three sintering temperatures (350, 380, and 400 °C) and times (60, 90, and 120 min) according to Taguchi orthogonal design of experiments for optimizing parameters using a Tubular furnace under a controlled argon gas atmosphere, as shown in Table 3. A two-step sintering technique was implemented. Before sintering at the desired time and temperature, samples were heated for 60 min at 450 °C, with an increment of 2 °C/min to ensure the decomposition of PMMA in the composites without distorting the composite. The sintered samples with a size of 10 mm × 10 mm (1 cm × 1 cm) developed in this study are shown in Figure 2. The porosity and relative density of the porous Al composites were computed via Archimedes’ principle using the following equation [45]:

(1) $$\mathrm{Porosity},\;P=\frac{W_{\mathrm{SS}}-W_{D}}{W_{\mathrm{SS}-}W_{S}}\;\times \;\rho_{H2O}$$

W_D_ is the dry (unsaturated) weight of the porous sample, W_SS_ is the saturated weight (assuming all pores were filled with liquid), and W_S_ is the saturated sample weight when submerged in liquid.

(2) $$\mathrm{Relative}\;\mathrm{Density}=\frac{\rho^{*}}{\rho_{s}}$$

where $\rho^{*}$ is the density of porous Al composite and $\rho_{s}$ is the theoretical density of Al (2.7 g/cm^3^).

#### 2.1.2. Characterization and Testing

The characteristics of the cross-sectioned surface of the developed Al foam composite were examined using a scanning electron microscope (SEM) and electron dispersive X-ray (EDX). The surface morphology, topography, and elemental composition associated with the matrix and reinforcement reaction were observed. The SEM result using the JEOL JSM-6300F (Tokyo, Japan) was used to determine the nature of particle distribution, particle size variation, and surface morphology of the Al foam composite samples. Archimedes’ principle was applied to measure the porosity and density of porous samples. Uniaxially compressive tests were performed on porous composite samples with a crosshead speed of 0.5 mm/min at room temperature and load cell of 30 kN (Dartec model 3500 universal testing machine). For the compressive response analysis, the average of three samples was determined. The area under the stress–strain curves determined the energy absorption capacity (W) of the resulting porous Al composites using the following equation [46]:

(3) $$W=\int_{0}^{\varepsilon }\sigma \;d\varepsilon$$

where σ and ε are the compression stress and strain, respectively.

### 2.2. Design of Experiments (DOE)

DOE is a statistical analytic tool that helps to detect the relevant factors for the fabrication process involving planning and experimentation [47]. Such methods allow the designer to control the changes by analyzing the responses through limited test runs. In our study, tests were performed using an L_9_ orthogonal array to acquire potential response factors influencing the input variables. Researchers have applied this concept and demonstrated its effectiveness in optimizing the properties of composites. Therefore, the DOE method was chosen for optimizing the compressive properties using various processing parameters in this study.

#### Taguchi’s L_9_ Orthogonal Array

Taguchi’s L_9_ orthogonal array is a planning and organizing tool for the selected parameters and their levels. The current investigation assumed that the problem was linear. The robust L_9_ orthogonal array design variables applied in this study included sintering temperature, compaction pressure, and sintering time. As shown in Table 3, the first column is the sintering temperature, followed by the compaction pressure and the sintering time. Table 4 shows a well-organized typical L_9_ orthogonal array with all possible combinations. Nine experiments were carried out based on the selected parameters and their levels. Following the tests, the two-response factors, plateau stress and energy absorption capacity, were obtained, as shown in Table 4. These values are vital for determining all the effects of running an L_9_ orthogonal array. However, the current work focused on the primary regression and ANOVA results, and the model is described as follows:

*Y* = *b*_0_ + *AX*_1_ + *BX*_2_ + *CX*_3_ (4)

where *Y* represents the dependent variable (plateau stress and energy absorption capacity), *b*_0_ is the intercept or reaction variable, and *A*, *B*, and *C* are the sintering temperature, compaction pressure, and sintering time, respectively.

If the cost and time allow, an L_9_ orthogonal array is preferred because more parameters of each level can be tested. The current study tested all three levels of the other parameters. The design is primarily used in industrial applications to investigate the impact of various control factors. In this type of investigation, the columns for the independent variables are orthogonal to one another. Thus, multiple levels and factors must be identified to describe an orthogonal array. Degrees of freedom (DF) are calculated for the three parameters in each of the three groups at several levels.

## 3. Results and Discussion

The processing techniques and process parameters significantly influence the performance of composites. In the powder metallurgy technique, the sintering temperature, compaction pressure, and sintering time are found to have more impacts on the properties of resultant porous composites [42]. Thus, the analysis of porous Al composites at varying sintering temperatures, compaction pressure, and sintering time was carried out and their effect on the compressive properties was evaluated. These properties are also associated with the morphology of these composites. The microporosity and distribution of Ti-coated diamond and PMMA particles are crucial for enhanced properties. The parameters chosen in this work reflected that the effect of sintering temperature was more on the morphology and the compressive properties followed by sintering time. Conversely, the effect of compaction pressure was least significant.

### 3.1. Morphological Analysis

**Figure 3:**
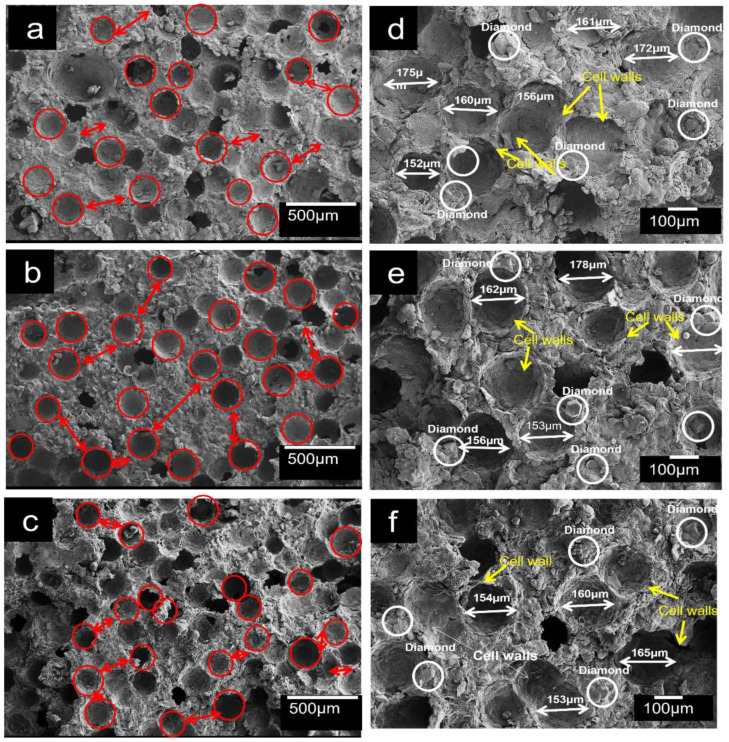
SEM morphology of porous Al composites developed at, (**a**,**d**) 580 °C, 350 MPa, and 60 min, (**b**,**e**) 590 °C, 350 MPa, and 120 min, and (**c**,**f**) 600 °C, 350 MPa, and 120 min.

**Figure 4:**
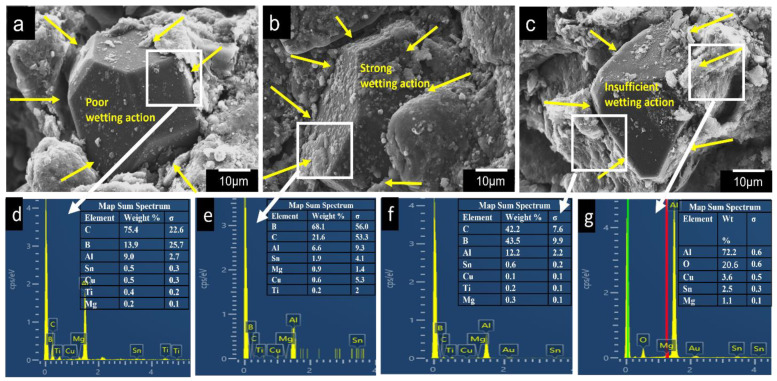
SEM images and EDX analysis of porous Al composites showing wetting action near the vicinity of diamond particles at different sintering temperatures, (**a**,**d**) 580 °C, (**b**,**e**) 590 °C, and (**c**,**f**,**g**) 600 °C.

The SEM micrographs of the porous Al composites reinforced diamond particles developed at varying sintering temperatures, compaction pressures, and sintering time were analyzed. Figure 3a–f shows the SEM images of porous composites at varying sintering temperature and time, at constant compaction pressure of 350 MPa. As evident from the SEM micrography, the composites exhibited closed macropores (Figure 3a–c) with the pore size ranging from 153–178 μm (Figure 3d–f, almost similar to the space holders (PMMA particles)). The pore shape was also similar to the shape of the PMMA particles, and is more prominent in the case of Figure 3b,e. Thus, this indicates the possible control of morphology of porous Al composites by selecting PMMA particles with suitable sizes and percentages. The control of the total porosity and pore size distribution in porous composites is essential for tailor-made materials with properties suited to particular applications [10,48,49]. Similar pore structures were obtained by Tan et al. [40], in porous Mg composites using PMMA particles as space holders. The macropores in the porous Al composite were uniformly distributed and separated by a unique cell wall, as shown in Figure 3. Figure 3 also shows that the composite fabricated at sintering temperature of 590 °C exhibited pores of well-defined cell walls, with greater cell wall thickness. While in case of porous Al composites sintered at 600 °C, the oxidation takes place as evident from Figure 4c,g. The formation of oxides at a higher sintering temperature of 600 °C during sintering reduces the strength of resultant composites and deteriorates their properties [50,51].

### 3.2. Effect of Sintering Temperature

The inadequate removal of space holders promotes crack formation in samples during sintering. To avoid this, the selection of proper sintering temperature is crucial for the development of porous Al composites using the space holder technique. Additionally, density and porosity depend on the sintering temperature. On the other hand, the sintering of Al at higher temperatures is detrimental, can lead to partial melting, oxidation, and evaporation of alloying or additive powders [52]. In this study, the porous composites were first heated at 450 °C to remove PMMA particles followed by sintering at 580 °C, 590 °C, and 600 °C. Similarly, researchers have employed the same temperature range to develop porous Al composites; however, they could not achieve better results beyond 580 °C [53]. Thus, the effect of sintering temperature and the optimum sintering temperature for porous composites reinforced with Ti-coated diamond particle was explored. Although the amount of PMMA particles was fixed at 30 wt.%, still the porosities achieved were more than 30% as mentioned in Table 5. This distinction is primarily due to the presence of microporosity in the composites. It can be observed that increasing the sintering temperature and time increases the microporosity of the Al composites as evident from Figure 4 and also illustrated by Table 5. To develop high-strength porous composites, the microporosity in a sintered sample should be as low as possible, otherwise they act as crack initiators. Thus, the Al porous composites with higher microporosity yield lower compression properties, as evident from Table 5.

The compressive properties of the composites improved up to a sintering temperature of 590 °C; however, a slight decline was observed at 600 °C. The plateau stress in the composites increased from 22.02 MPa (at 580 °C) to 31.06 MPa (at 590 °C) and shifted to 30.02 MPa (at 600 °C). In addition, the energy absorption capacity of sintered porous Al composites enhanced from 5.77 Mj/m^3^ to 9.19 Mj/m^3^, indicating an improvement of about 1.6 times. The improvement in compressive properties can be attributed to the addition of tin, boron, and magnesium additives to Al that promotes the liquid phase sintering and leads to improved interfacial bonding between matrix and reinforcement (Ti-coated diamond) as evident from Figure 4. Similar results were achieved in porous composites by using Sn, Mg, and boron as additives for liquid phase sintering [11,14]. Figure 4 shows the SEM and EDX of the Al alloy matrix and diamond interface, where carbon content shows the presence of diamond particle and other elements are the Al and the additives. In porous composite sintered at 580 °C, there is a poor wetting action due to absence of sufficient liquid phase required to bond diamond interface with the Al alloy matrix interface. Further, in case of porous Al composites sintered at 590 °C, there exists a strong wetting action due to the presence of a good amount of additives that are mostly in liquid phase, as evident from Figure 4e, thereby improving the bonding strength of Ti-coated diamond in the Al alloy matrix. This leads to enhanced properties, primarily due to the low viscosity wetting liquid phase; as the temperature rises above its melting point, the viscosity decreases exponentially. However, the porous composites sintered at 600 °C exhibited insufficient wetting action as a result of oxidation at a high temperature. This results in deterioration of compressive properties.

### 3.3. Effect of Compaction Pressure

**Figure 5:**
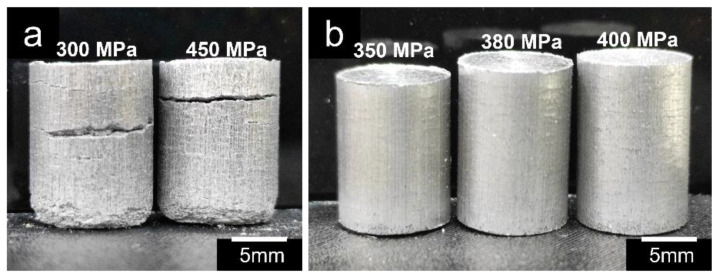
Samples compacted at (**a**) 300 and 450 MPa, and (**b**) at 350, 380, and 400 MPa.

Compaction pressure influences the bonding strength between the Al alloy matrix and reinforcement particles in the resultant green compacts. The presence of oxide film on the surface of Al particles prevents strong binding between them; therefore, the film must be broken down by applying adequate compacting pressure. This procedure brings fresh metallic surfaces of particles into contact for sintering [54]. Although the effect of compaction pressure on compressive properties was least significant, it still has some impact on the morphology and powder packing. It was discovered that compaction pressures less than 350 MPa resulted in defective samples with inadequate green strength to endure additional processes and examinations (Figure 5a). On the contrary, pressures above 400 MPa resulted in the crushing of PMMA particles, leading to crack formation and alteration in the final morphology and pore size of the composite. The results demonstrate that a compaction pressure between 350–400 MPa produced better outcomes as the composites retained their original shapes with sharp edges, with no collapsing of PMMA particles, and exhibited sufficient strength for subsequent processing steps.

The compaction pressures ranging from 300 to 400 MPa are considered high compared to the conventional pressures used in the PM technique [54]. The compaction stress gets distributed among components, including diamond particles and PMMA particles in the porous Al composites, thus higher compaction pressures are required to obtain good compacting. However, higher pressures can distort the shape of PMMA particles. To prevent this, the pressure was reduced by using binders, i.e., CLE-safe oil was added to the powder mixture [55]. However, such addition may incur side effects as it increases the cost of the process, releases contaminating gases, and involves a time-consuming mixing process.

**Figure 6:**
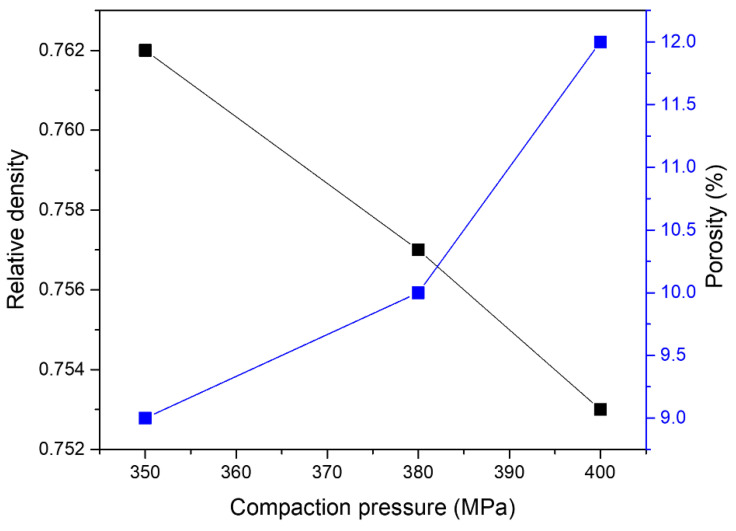
Effect of compaction pressure on relative density and porosity of Al compacts.

**Figure 7:**
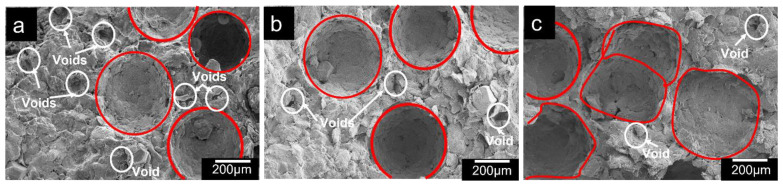
SEM micrography of porous Al composites at sintering temperature of 590 °C and at varying sintering time (**a**) 350 MPa, 120 min, (**b**) 380 MPa, 90 min, and (**c**) 400 MPa, 60 min (Red circles represent pore shapes and the voids are encircled by white circles).

In addition, the spherical shape of PMMA particles allow easy movement and rearrangement of these particles during the compaction. This property resulted in uniform macropore distribution in the green compacts and improved PMMA particle interconnectivity [56]. In fact, when compared to other space-holder materials such as NaCl [43,57], the spherical pores formed by spherical PMMA particles have reduced surface roughness with fewer corners and edges, which helps to reduce local stress concentrations and non-uniform deformation during compaction, and thus improves the strength of porous Al composites. Further, the compaction pressure also influences the relative density and porosity, as shown in Table 5. The relative densities of porous Al compacts (shown in Figure 5b) decreased with the increase in compaction pressure while the porosities increased, evident from Figure 6. This can be attributed to the deformation of PMMA particles on application of higher compaction pressure, where PMMA particles exceed their elastic limit and deform, or may crack leading to formation of distorted pore morphology as shown in Figure 7 and cracks in cell walls [58].

### 3.4. Effect of Sintering Time

After sintering temperature (Table 4), sintering time is also crucial for determining the properties of porous composites. Providing sufficient time for sintering promotes effective diffusion, easy and fast permeation, and improved bonding between different constituents in the composites, which eliminates microporosity. The plateau stress and energy absorption capacity increased on sintering from 60–90 min and decreased with increasing sintering time to 120 min. There occurs the filling of voids by liquid metals during liquid sintering from 60 to 90 min, thereby decreasing the microporosity and improving the compressive properties. The diffusion rate increases with the increase in sintering time, and the Al alloy matrix powder merges together by forming necking, resulting in a closed porosity and denser structure [59]. However, when the sintering time increases, small pores coalesce, resulting in the formation of larger pores (voids), which slows the sintering process. The formation of large voids in porous structure reduces their compressive properties. A similar effect was observed for porous steel composites developed using the PM technique by Tatt et al. [60]. Figure 7 shows an SEM image of pore coalescence (voids) at sintering times of 60, 90, and 120 min, mostly in the case of the porous composite with 120 min sintering time (Figure 7a). As a result, longer sintering times can adversely affect the composite’s strength.

### 3.5. Analysis of Signal-to-Noise (S/N) Ratio

**Figure 8:**
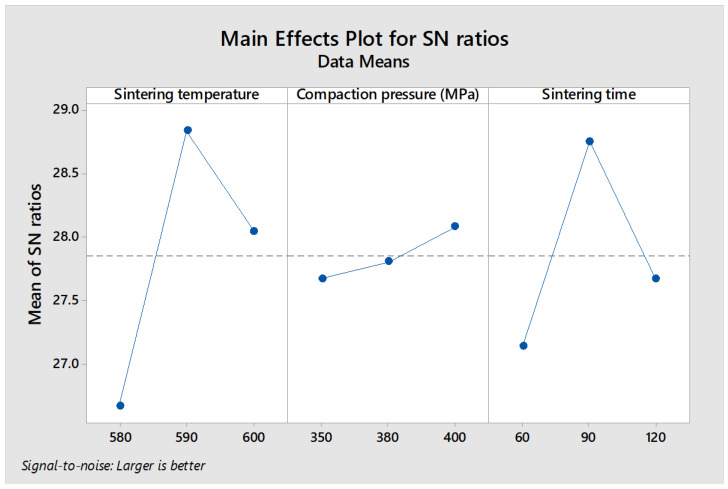
S/N ratio response curve of plateau stress.

**Figure 9:**
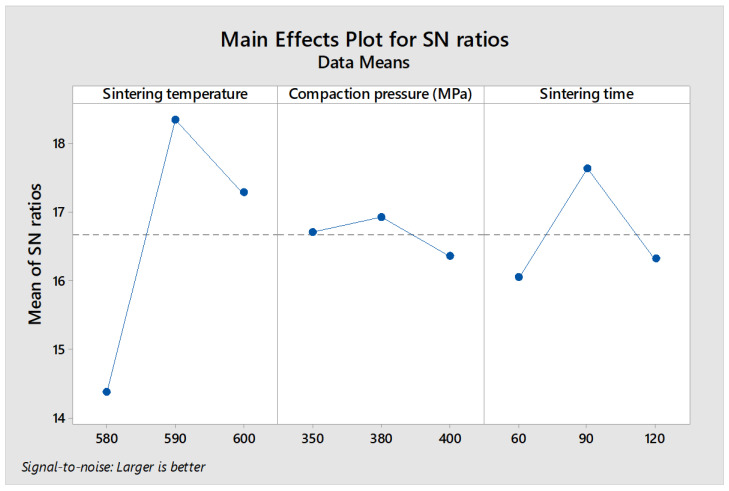
S/N ratio response curve of energy absorption capacity.

The response variables, including plateau stress and energy absorption capacity, were considered to ensure improved compressive properties. In practical applications, the plateau stress and energy absorption capacity should be maximized to increase applicability. Therefore, the rule of thumb for determining the signal-to-noise (S/N) ratio for the response factors is “the larger, the better”. Figure 8 and Figure 9 show the plots of the main effect that explains the influence and impact of input process parameters on plateau stress and energy absorption capacity, respectively. Figure 8 presents the S/N analysis of plateau stress, with the sintering temperature at level 2 (590 °C), compaction pressure at level 3 (400 MPa), and sintering time at level 2 (90 min), resulting in maximum plateau stress. Meanwhile, the maximum energy absorption capacity was achieved at sintering temperature (590 °C), compaction pressure (380 MPa), and sintering time (90 min) of level 2, as shown in Figure 9.

### 3.6. Analysis of Variance (ANOVA)

**Figure 10:**
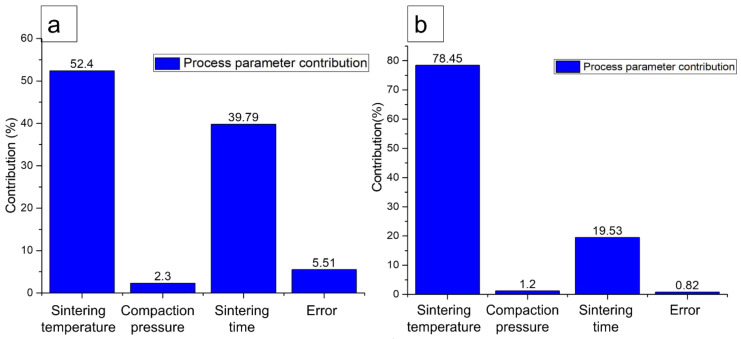
Percentage of the contribution of process parameters on (**a**) plateau stress and (**b**) energy absorption capacity.

The effect of parameters and their relationships were investigated using ANOVA by comparing the mean square against response errors at predetermined confidence levels. The ANOVA helps to determine the impact of each factor on the overall result variance. Table 6 and Table 7 show the ANOVA results for both response factors (plateau stress and energy absorption capacity). The analysis was conducted with a 10% significance level corresponding to a 90% confidence level. Each ANOVA table includes a percentage contribution of each factor’s variable to the total variation. The contribution of each factor that impacts response factors (plateau stress and energy absorption capacity), as shown in Figure 10, were analyzed based on the results from the ANOVA. The assigned variable is statistically significant if the F-value exceeds 8%. Table 6 and Figure 10a show that sintering temperature produced the highest significant impact (52.40%) on plateau stress, followed by sintering time (44.729%), and compaction pressure (2.3%). Similarly, Table 7 and Figure 10b show that sintering temperature produced the highest significant impact (78.45%) on energy absorption capacity, followed by a sintering time (19.53%), and compaction pressure (1.2%).

The compaction pressure, sintering temperatures, and sintering time tested in this study were selected based on the literature. The strength of a composite can be increased by forming a strong interface bond between the matrix and the reinforcements. Such formation can be accomplished by selecting the appropriate processing parameters, such as compaction pressure, sintering temperature, and sintering time. The compaction process holds together the neighboring powder particles by forming cold welds, which impart adequate green strength to the compacts for enduring further processes. Meanwhile, the sintering time affects the diffusion rate and the grain growth of the composites. A sintering temperature below the melting point of the major component of the composite enables optimum grain growth, adequate to fill the voids and strengthen the composites. Sintering temperature influences the microporosity of the composites, thus significantly impacting their strength [61]. The impact is evident in the significant effect of the sintering temperature on the plateau stress (52.4%) and energy absorption capacity (78.45%) observed in this study.

The effect of the compaction pressure was minimal and remained at 2.3% and 1.2% for plateau stress and energy absorption capacity, respectively. The plausible explanation is the restriction imposed on compaction pressure to prevent the distortion or crack of PMMA particles. Therefore, the parameters selected in this study mainly exhibited the least change in the porosity of green compacts, as shown in Figure 6.

The linear polynomial model below describes the plateau stress and energy absorption capacity as a function of sintering temperature, compaction pressure, and sintering time obtained from the analysis of variance. For plateau stress and energy absorption capacity, the greatest R^2^ predictions were determined to be 94.49% and 99.18%, respectively, indicating that the model can predict new observations nearing or matching the sample data. Furthermore, this study can be explained well by the regression equation.

### 3.7. Regression Analysis

The L_9_ orthogonal array was used with MINITAB software to recognize a statistical model based on linear regression equations. A linear polynomial model (regression equations) for plateau stress and energy absorption capacity based on each factor was obtained and presented in the equations below:

Plateau stress = 24.926 − 3.372 A1 + 2.821 A2 + 0.551 A3 + 1.17 B1 − 1.83 B2 + 0.668 B3 − 2.116 C1 + 3.77 C2 − 1.66 C3(5)

Energy absorption capacity = 6.9733 − 1.720 A1 + 1.337 A2 + 0.383 A3 + 0.534 B1 − 0.344 B2 − 0.190 B3 − 0.570 C1 + 1.096 C2 − 0.526 C3(6)

where A is the sintering temperature, B is the compaction pressure, and C is the sintering time.

From the linear regression Equations (5) and (6), response plateau stress and energy absorption values were calculated to find the percentage deviation. The significant residual errors of each test, and the maximum errors of 1.40 and 1.37 (Table 8 and Table 9) were obtained for plateau stress and energy absorption capacity, respectively.

**Figure 11:**
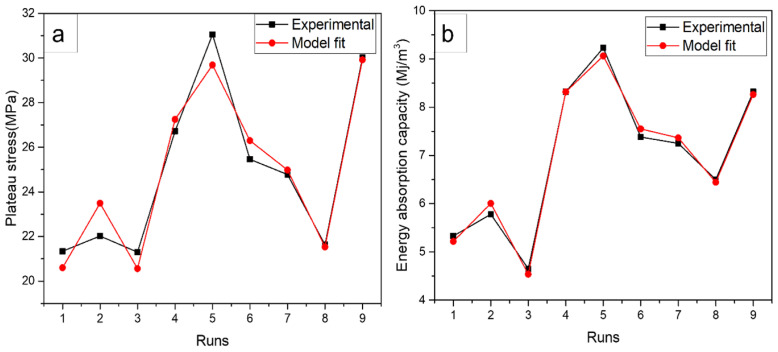
Comparison of experimental and modal predicted values for, (**a**) plateau stress and (**b**) energy absorption capacity.

The data for each experiment run are illustrated in Figure 11, which shows the discrepancy in the experimental and model fit values. The device errors and the processing errors in the measurement system indicate the rate of change in plateau stress and energy absorption capacity. The experimentation also relied on other factors, including mixing parameters and composition, which also influenced the properties of the composites.

### 3.8. Response Optimization

**Figure 12:**
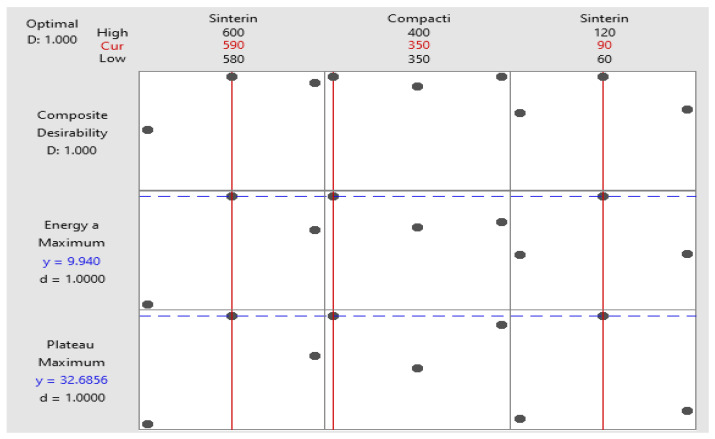
Optimization plot for plateau stress and energy absorption capacities (black point represent the output data, red lines join the maximum values of outputs obtained at some optimal parameters and blue line shows the optimal output values).

The response optimization study was carried out to obtain the best results and parameters, as shown in Table 10. Based on the previous investigations, the motivation for conducting this study was to investigate the control of the compressive properties of porous Al composites. Therefore, the goal was to maximize results to optimize the plateau stress and energy absorption capacity. The low plateau stress is presented in Table 10, which can be regarded as the predicted value. Although maximum and minimum variations exist for the target and lower values, they are impractical. The compressive properties also depend on the composition, mixing parameters, crosshead speed during compression testing, and other parameters. It could result in extremely high or low values. Therefore, a safe and reasonable value for plateau stress and energy absorption capacity can be considered as the fit values as outlined in Table 10 and depicted in Figure 12, which can be attained at a sintering temperature of 590 °C, compaction pressure of 350 MPa, and sintering time of 90 min.

### 3.9. Contour Plot

**Figure 13:**
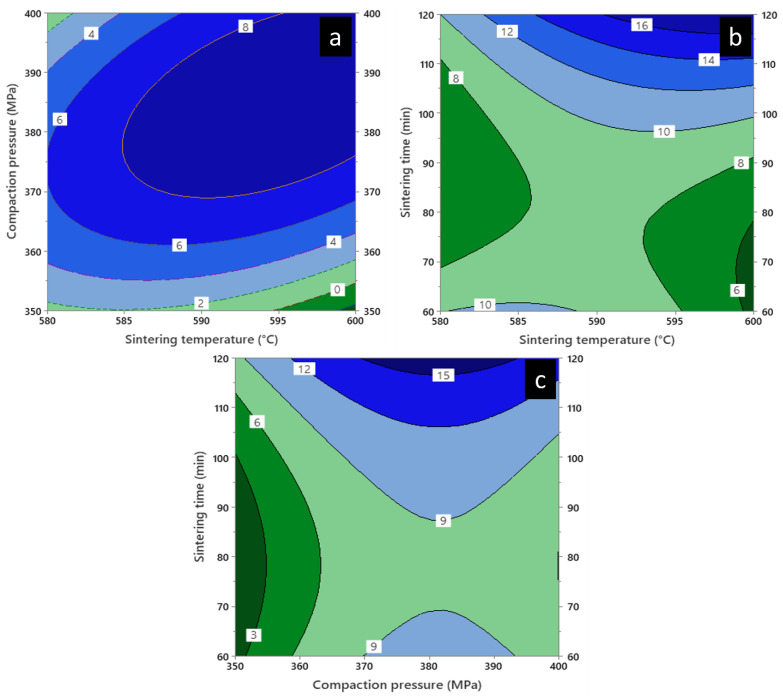
Contour plot of plateau stress in diamond-reinforced porous Al composites showing, (**a**) Compaction pressure vs. sintering temperature, (**b**) Sintering time vs. sintering temperature and (**c**) Sintering time vs. compaction pressure.

**Figure 14:**
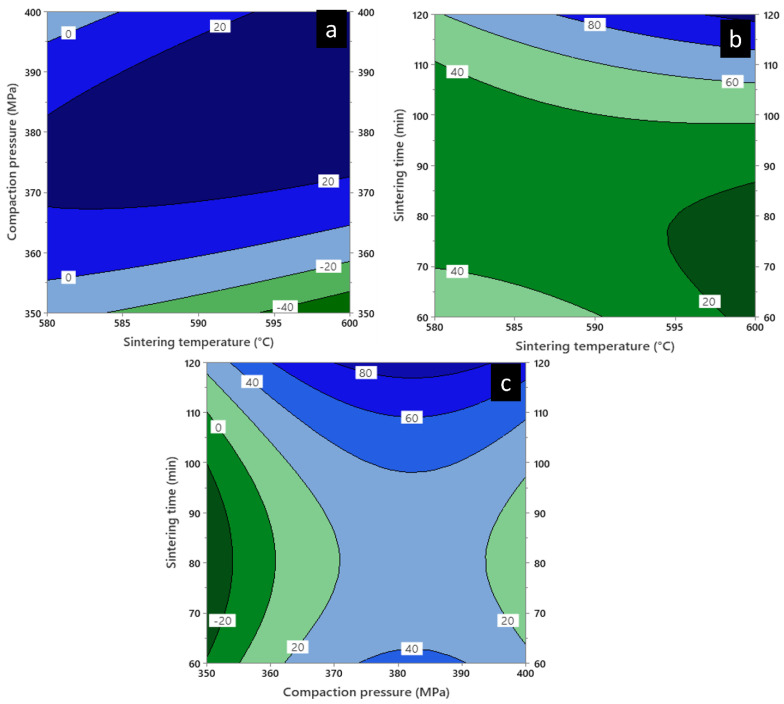
Contour plot of energy absorption capacity of diamond-reinforced porous Al composites showing, (**a**) Compaction pressure vs. sintering temperature, (**b**) Sintering time vs. sintering temperature and (**c**) Sintering time vs. compaction pressure.

Figure 13 and Figure 14 show the contour plot of the plateau stress and energy absorption capacity involving three different combinations of variables for diamond-reinforced porous Al composites: the influence of sintering time (90 min) in Figure 13 and Figure 14a, compaction pressure (375 MPa) in Figure 13 and Figure 14b, and sintering temperature (590 °C) in Figure 13 and Figure 14c on plateau stress and energy absorption capacity. In combination with the parameters shown in Figure 13a, the plateau stress increased with the increase in the sintering and decreased on increasing the compaction pressure. Additionally, Figure 13b shows that the maximum sintering temperature and time influenced the plateau stress, resulting in a sudden increment in the latter. However, Figure 13c shows an increase in the plateau stress with an increase in sintering time, with a medium compaction pressure. Meanwhile, Figure 13a shows a reduction in the plateau stress (−40 MPa) at a high sintering temperature and low compaction pressure.

A similar result was obtained for the energy absorption capacity as shown in Figure 14a. However, the energy absorption capacity was higher than the combined values of the sintering temperature and time as shown in Figure 14b. Also, Figure 14c shows the maximum energy absorption capacity was achieved at medium compaction pressure with maximum sintering time. Thus, the contour plots verified the impact of the selected parameters on changing the response factors.

### 3.10. Confirmation Test

**Figure 15:**
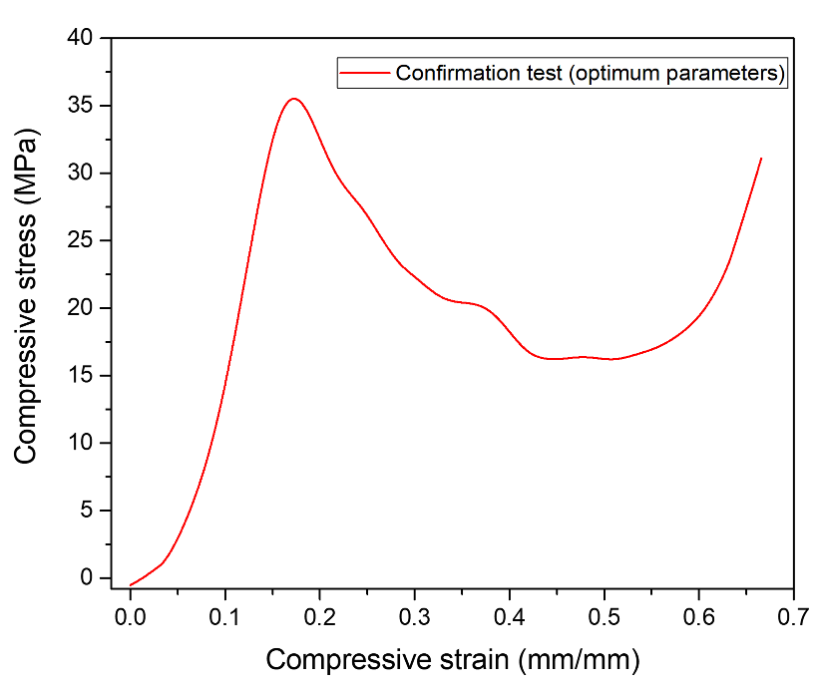
Compressive stress–strain diagram of confirmation test at optimum parameters.

In the DOE approach, the final step is the confirmation of experiments. After investigating the optimal test conditions, the confirmation was performed considering the optimum level of factors, sintering temperature of 590 °C, compaction pressure of 350 MPa, and sintering time of 90 min as evident from Table 10. The acquired results were eventually compared with the predicted results. Figure 15 shows the stress–strain diagram of the confirmation test from where responses are measured.

Further, Table 11 demonstrates the comparative results obtained using optimum parameters. It has been observed that there was reasonable agreement between the experimental and predicted results. However, errors of −10.5% for plateau stress and 6.6% energy absorption capacity were observed.

Finally, to evaluate the contribution of this work in improving the properties of porous Al composites, the compressive properties obtained in this study are compared to those found in other studies and are presented in Table 12. The comparison shows that the Ti-coated diamond particles contribute more to the enhancement of plateau stress and energy absorption capacity in this work than by most of the referenced reinforcements in other studies [4,39,62,63,64,65]. As a result, the parameter optimization enabled development of porous Al composites with a well-defined porous structure using the powder metallurgy approach resulted in significant improvement in compressive properties of the Al composite foams.

## 4. Conclusions

Using experimental, numerical, and optimization methods, this study investigated the effect of powder metallurgy processing parameters on the compressive properties of porous Al composites. The developed composites had a spherical porous structure with Ti-coated diamond particles distributed uniformly within the Al matrix alloy, according to their morphology. Furthermore, the Ti-coated diamond particles were well bonded with the Al matrix alloy, revealing improved wettability due to the coated diamond particles and the inclusion of additives such as Mg, Sn, Cu, and B, which promoted liquid sintering. The densities and porosities of composites were also affected by parameter variations. The plateau stress and energy absorption test results revealed that the highest values were obtained for a sintering temperature of 590 °C, a compaction pressure of 380 MPa, and a sintering time of 90 min. However, Taguchi’s L_9_ orthogonal array was used to obtain the optimal parameters required to improve the plateau stress and energy absorption capacities.

Statistical and regression analyses, model prediction, and contour plots were used to examine the effect of process parameters on compressive properties. The linear regression equation values were compared to the experimental test results. Sintering temperatures of 590 °C, compaction pressures of 350 Mpa, and sintering times of 90 min were the response optimized results. Finally, the results were validated by running the confirmation tests under optimized conditions. The model was found to be reliable and significant, with the lowest percentage deviation in plateau stress and energy absorption capacity. The current study’s findings agree well with the marginal discrepancy of 10.5% in plateau stress and 6.6% in energy absorption capacity values. This disparity can be attributed to other factors, such as other processing parameters and varying compositions, all of which influence compressive properties. As a result, it is critical to evaluate the results for different compositions and other factors. In order to improve the properties, the compressive properties can be further optimized by varying the Ti-coated diamond content and the PMMA content and size.

## Figures and Tables

**Table 1:** Particle content, average particle size, and purity of powders for sample preparation.

Material	wt.%	Average Particle Size (µm)	Purity (%)
Alloy matrix			
Al	94	45	99.9
Mg	1	10	99.9
Sn	2	45	99.5
Cu	2	75	99.5
B	1	10	99.5
Reinforcement			
Ti-Diamond	5	45	99.5
Space holder			
PMMA	30	150	99.9

**Table 2:** Powder metallurgy processing parameters.

Process		Parameters
Mixing	Metallic mix (MM)	300 rpm, 24 h
	Mixing (MM + diamond)	800 rpm, 2 h
	Mixing (MM + diamond + PMMA)	800 rpm, 2 h
Compaction Pressure		350, 380, and 400 MPa
Sintering temperature		580, 590, and 600 °C
Sintering time		60, 90, and 120 min

**Table 3:** Control factors (process parameters) and their levels.

Factors	Symbol	Unit	Level 1	Level 2	Level 3
Sintering temperature	A	°C	580	590	600
Compaction pressure	B	MPa	350	380	400
Sintering time	C	min	60	90	120

**Table 4:** L_9_ orthogonal array layout with design factors.

Runs	A	B	C	Plateau Stress (MPa)	Energy Absorption Capacity (Mj/m^3^)
1	580	350	60	21.34	5.33
2	580	380	90	22.02	5.78
3	580	400	120	21.30	4.65
4	590	350	120	26.72	8.32
5	590	380	90	31.06	9.23
6	590	400	60	25.46	7.38
7	600	350	120	24.78	7.25
8	600	380	60	21.63	6.50
9	600	400	90	30.02	8.32

**Table 5:** Density and porosity of porous Al composites for nine sets of parameters.

S.No	1	2	3	4	5	6	7	8	9
Porosity (%)	40	40	41	41	42	41	43	44	43
Relative density	0.83	0.81	0.80	0.86	0.84	0.86	0.84	0.83	0.84

**Table 6:** ANOVA Variance table for plateau stress.

Source	DF	Seq SS	Contribution	Adj SS	Adj MS	F-Value	*p*-Value
Sintering temperature	2	58.903	52.40%	58.903	29.451	9.51	0.095
Compaction pressure	2	2.589	2.30%	9.497	4.748	1.53	0.395
Sintering time	2	44.729	39.79%	44.729	22.364	7.22	0.122
Error	2	6.194	5.51%	6.194	3.097		
Total	8	112.415	100.00%				

**Table 7:** ANOVA Variance table for energy absorption capacity.

Source	DF	Seq SS	Contribution	Adj SS	Adj MS	F-Value	*p*-Value
Sintering temperature	2	14.6761	78.45%	14.6761	7.33803	95.91	0.010
Compaction pressure	2	0.2245	1.20%	0.8049	0.40243	5.26	0.160
Sintering time	2	3.6545	19.53%	3.6545	1.82723	23.88	0.040
Error	2	0.1530	0.82%	0.1530	0.07651		
Total	8	18.7080	100.00%				

**Table 8:** Fits and diagnostics for all observations for plateau stress.

Runs	Experimental	Model Fit	SE Fit	Resid	Std Resid	Del Resid
1	21.34	20.60	1.68	0.74	1.40	8.65
2	22.02	23.49	1.41	−1.47	−1.40	−8.65
3	21.30	20.56	1.68	0.74	1.40	8.65
4	26.72	27.25	1.41	−0.53	−0.51	−0.39
5	31.06	29.69	1.41	1.37	1.31	2.43
6	25.46	26.30	1.55	−0.84	−1.01	−1.02
7	24.78	24.98	1.41	−0.20	−0.19	−0.14
8	21.63	21.53	1.68	0.10	0.19	0.14

**Table 9:** Fits and diagnostics for all observations for energy absorption capacity.

Runs	Experimental	Model Fit	SE Fit	Resid	Std Resid	Del Resid
1	5.330	5.217	0.264	0.113	1.37	3.74
2	5.780	6.005	0.222	−0.225	−1.37	−3.74
3	4.650	4.537	0.264	0.113	1.37	3.74
4	8.320	8.318	0.222	0.002	0.01	0.01
5	9.230	9.062	0.222	0.168	1.02	1.04
6	7.380	7.550	0.244	−0.170	−1.30	−2.38
7	7.250	7.365	0.222	−0.115	−0.70	−0.56
8	6.500	6.443	0.264	0.057	0.70	0.56
9	8.320	8.263	0.264	0.057	0.70	0.56

**Table 10:** Response optimization: processing parameters.

Response			Goal	Lower	Target	Upper	Weight	Importance
Energy absorption capacity (Mj/m^3^)			Maximum	4.65	9.23		1	1
Plateau stress (MPa)			Maximum	21.30	31.06	------	1	1
**Solution**								
Solution	Sintering temperature (°C)	Compaction pressure (MPa)	Sintering time (min)		Energy absorption capacity Fit		Plateau stress Fit	Composite Desirability
1	590	350	90		9.94		32.6856	1
Response			Fit	SE Fit	95% CI		95% PI	
Energy absorption capacity (Mj/m^3^)			9.940	0.332	(8.510, 11.370)		(8.079, 11.801)	
Plateau stress (MPa)			32.69	2.12	(23.59, 41.79)		(20.85, 44.52)	

**Table 11:** Confirmation test comparisons with predicted values.

Responses	Prediction	Experimentation	Error (%)
Plateau stress (MPa)	32.69	36.12	−10.5
Energy absorption capacity (Mj/m^3^)	9.94	10.6	6.6

**Table 12:** Comparison of compressive properties obtained in this study with another research.

Matrix	Reinforcement	Porous Media	Method	Relative Density	Plateau Stress (MPa)	Energy Absorption Capacity (Mj/m^3^)	Ref.
AlSnCuMgB	Ti-Coated Diamond	PMMA	PM	0.86	36.12	10.6	[This Study]
AlSnMg	-	PMMA	PM	0.80	29.41	3.65	[39]
Al	B_4_C	Carbamide	PM	0.48	23.9	11.47	[4]
Al	-	Carbamide	PM	0.48	28.06	5.2	[62]
Al	SiC & MWCNT	Calcium hydride	LM	0.30	16.6	4.87	[63]
AlSiMgFe	SiC	Titanium hydride	PM	0.81	26.43	5.79	[64]
Al	CNT	Carbamide	PM	0.40	33.1	11.6	[65]

## Data Availability

Not applicable.
